# Accelerating the Gillespie Exact Stochastic Simulation Algorithm Using Hybrid Parallel Execution on Graphics Processing Units

**DOI:** 10.1371/journal.pone.0046693

**Published:** 2012-11-09

**Authors:** Ivan Komarov, Roshan M. D'Souza

**Affiliations:** Department of Mechanical Engineering, Complex Systems Simulation Lab, University of Wisconsin-Milwaukee, Milwaukee, Wisconsin, United States of America; University of Hertfordshire, United Kingdom

## Abstract

The Gillespie Stochastic Simulation Algorithm (GSSA) and its variants are cornerstone techniques to simulate reaction kinetics in situations where the concentration of the reactant is too low to allow deterministic techniques such as differential equations. The inherent limitations of the GSSA include the time required for executing a single run and the need for multiple runs for parameter sweep exercises due to the stochastic nature of the simulation. Even very efficient variants of GSSA are prohibitively expensive to compute and perform parameter sweeps. Here we present a novel variant of the exact GSSA that is amenable to acceleration by using graphics processing units (GPUs). We parallelize the execution of a single realization across threads in a warp (fine-grained parallelism). A warp is a collection of threads that are executed synchronously on a single multi-processor. Warps executing in parallel on different multi-processors (coarse-grained parallelism) simultaneously generate multiple trajectories. Novel data-structures and algorithms reduce memory traffic, which is the bottleneck in computing the GSSA. Our benchmarks show an 8×−120× performance gain over various state-of-the-art serial algorithms when simulating different types of models.

## Introduction

Mechanistic modeling of biological systems requires the simulation of complex sets of interconnected chemical reaction channels. There are two fundamental approaches to simulating such systems: deterministic and stochastic. Deterministic approaches are applicable when the number of molecules of the reactants is large enough such that the state of the system can be tracked in terms of the concentrations of the reactants with real numbers. The time evolution of the system state is governed by a set of non-linear coupled differential equations. In the case of a low number of reactant molecules, the deterministic approaches fail and stochastic approaches that track the actual number of reactant molecules have to be used. The processes that occur in such bio-chemical systems can be modelled using the Chemical Master Equation (CME) [1], which describes the time evolution of the probability density function (PDF) of the system state. Since analytical and numerical solutions are challenging, Monte Carlo schemes that sample instances of the underlying stochastic processes are used. The Gillespie Stochastic Simulation Algorithm (GSSA) [2] and its variants [3], [4] are the most popular Monte Carlo schemes that are used to solve the CME.

The exact GSSA and its variants [2], [4]–[8] advance the system state by executing one reaction at a time. In cases where the simulations involve whole cells or cell colonies where the total number of reaction channels can approach 10^5^–10^6^, even simulation of a single trajectory becomes prohibitively expensive. Moreover, since a single simulation is just one sample trajectory in what is essentially a probability distribution, dense sampling (simulation of 10^5^–10^6^ of trajectories) is needed to generate a dense dataset to back calculate the time evolution of the PDF. Both of these issues render the simulation of biologically relevant models intractable.

The computational intractability of the GSSA has been addressed in several ways. The approximation approaches such as the *τ*-Leaping method and its variants [3], [9]–[12] use an approximation under certain assumptions that allows the advancement of the system state by several reactions within the given time step *τ*. Improved algorithms for the exact method such as the Optimized Direct Method (ODM) [5], the Next Reaction Method(NRM) [4], the Sorting Direct Method (SDM) [7], and the Logarithmic Direct Method (LDM) [6] speed up computation of the altered system state and the search process of the next reaction to be fired. One of the most interesting algorithms is the Partial-propensity Stochastic Simulation Algorithm (PSSA) method which uses propensity factoring and grouping of reactions that share common reactants into a special data structure to reduce the amount of computations needed to handle reaction firing [13]. There are two variants of the PSSA algorithm: one is the PSSA-CR, that uses composition rejection (CR) [14] to select the next reaction to be fired. The other is the Sorting Partial-propensity Direct Method (SPDM), which uses the SDM mechanism to search for the next reaction to be fired.

Another approach is through leveraging the power of parallel computing. Coarse-grained parallelization is used to generate ensemble averages by running several concurrent trajectories independently on different computing cores. Such parallelization has been attempted on clusters [15], multi-core CPUs [16] and on graphics processing units [17], [18]. In coarse-grained parallelization of the Direct Method on GPUs [17], each GPU thread runs one realization, i.e., each thread executes the serial algorithm. The limited size of share memory (user controlled cache) means that the model size is limited to *N*+*M*<63 where *N* is the number of reactions and *M* is the number of reactants for the most efficient thread configuration. Fine-grained parallelization of the First Reaction Method (FRM) has been attempted [19]. However, this implementation is limited by the constant data transfer between CPU and GPU because both devices are involved in processing. An interesting alternative is hardware implementation on Field Programmable Gate Arrays (FPGAs) [20]. However, the limited size of the programmable hardware limits the size of networks to less than 10^4^ reactions.

### Graphics Processing Units

Graphics Processing Units (GPUs) were initially built to speed up the rendering of 3-D images for real-time display. GPUs follow the data-parallel computing model where the same set of instructions is applied to a large data set whose individual elements are of the same type. However, the same computing model is applicable to a wide variety of scientific problems. The development of direct APIs [21], [22] specifically for use in scientific computing has enabled the acceleration of many such problems and has led to an entirely new area in high performance computing commonly known as General Purpose Graphics Processing Units (GPGPU) [23].

**Figure 1:**
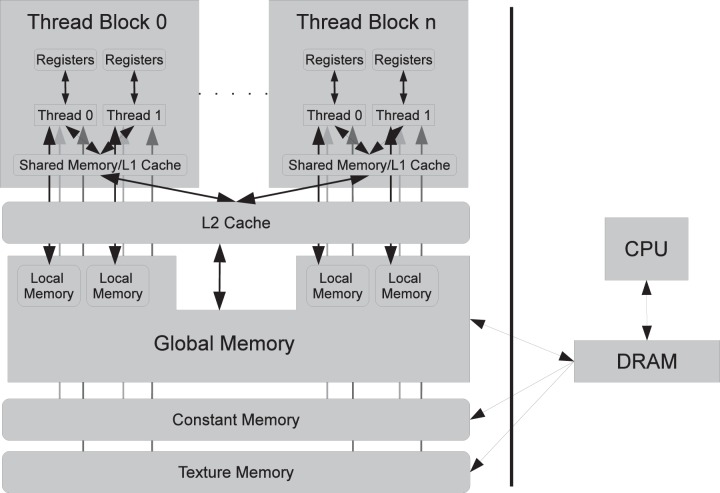
CUDA Computing Model. The basic execution unit is a thread. Threads are grouped into thread blocks. Each thread block is executed on a single multi-processor. Threads in a thread block can communicate through shared memory which is essentially a user-controlled cache. In the latest GPUs, shared memory can be configured to act as an L1 cache. Register space can be used to store data local to threads with the fastest access speed. Spill-over data that does not fit in registers goes to local memory, which is physically stored in the main memory and which is very slow. Main memory has three components. Global memory is accessible to all threads and is cached through an L2 cache that is shared among all multi-processors on the latest generation GPUs. Constant memory is typically used to store data that is used by all threads (simulation constants) and that is automatically cached. Texture memory is read-only memory with automatic cache.

In this paper we will describe the architecture of NVIDIA GPUs as this implementation is specific to this vendor. The GPU computing cores are organized into a set of multi-processors. Each multi-processor contains several serial processors that share instruction dispatch. There are several types of memories available (Figure 1). Multi-processors have registers and on-chip memory that can be configured as a user-controller cache called shared memory or as an automatic L1 cache. In addition, the latest Fermi architecture has an L2 cache shared between all multiprocessors. The device/global memory is off-chip and is equivalent to random access memory on the CPU. Furthermore, regions in the global memory can act as read-only texture and as constant memory. Both of these have an on-chip automatic cache, although the constant memory cache has a smaller latency. Constant memory is typically used to store system constants that will be accessed by all threads. Texture memory can be used to lower the overhead of irregular global memory access patterns.

NVIDIA has made available an API called compute unified device architecture (CUDA) [24], [25], which provides an abstract programming model of the GPUs resources with a set of constructs that describes thread, memory hierarchies, and synchronization primitives, among other things. The host/CPU controls the execution of the *kernel*, which is a parallel program that executes on the GPU. The basic execution unit on a GPU is a thread. Threads are grouped into thread blocks (TBs). All threads in a TB are executed on a single multi-processor and therefore can communicate by using shared memory. At the hardware level, threads are grouped into *warps*. A warp is a grouping of 32 consecutive threads synchronously executing the same instruction on different cores of a single multiprocessor on different data pieces indexed by the thread ID. On the latest GPUs, if threads in a warp access memory in a contiguous 128 byte segment, it results in *coalesced* memory access, i.e., a single memory transaction. Furthermore, shared memory is mapped to 16 banks and if threads in a half warp access segments of shared memory that map to the same bank, bank conflicts will result, which degrades performance. A very useful set of functions that is available is *warp voting* functions that allow checking the status of a computation with respect to all threads in the warp.

### Gillespie Stochastic Simulation Algorithm (GSSA)

The GSSA is a Monte-Carlo simulation of a single trajectory from the chemical master equation of spatially homogeneous reacting systems. Consider a reaction system with a list of species *S* and a list of possible reaction channels *R* that describe the interaction between species. The reactions can be uni-molecular (*s_i_* →) (Type 1), bi-molecular (*s_i_*+*s_j_* →) (Type 2), or bi-molecular where *s_i_* = *s_j_* (Type 3). Given a initial species population 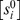, the algorithm proceeds as follows:

- 1 Set simulation time *t* = 0, initialize the reactant populations 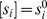.
- 2 For each reaction *r_l_* ∈ *R*, given rate constant *k_l_* compute propensities *a_l_* as

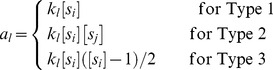



Compute the sum of propensities 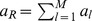.

- 3 Choose the reaction to be fired next *r_f_* such that

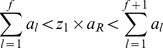
where *z*_1_ is a uniform random number in the unit interval.

- 4 Update species population due to firing reaction *r_f._*
- 5 Update simulation time *t_sim_* as

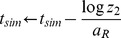
where *z*_2_ is a uniform random number in the unit interval.

- 6 Record populations [*s_i_*] of certain species.
- 7 If *t_sim_* < *t_f_* go to step 2.

The Optimized Direct Method maintains a reaction dependency graph. Since each reaction fired only affects a few other reactions, the dependency graph significantly reduces the cost of updating propensities. Instead of computing the total sum of propensities *a_R_* at each update step, it only computes the sum incrementally. The search for the reaction to be fired, however, is a simple linear search. Since in general bio-chemical systems only a small fraction of the reactions are regularly fired, sorting the list of propensities by magnitude significantly reduces the cost of the linear search. Therefore, ODM uses pre-simulation to sort the list of reactions based on average propensities over the course of the simulation. The Logarithmic Direct Method, in addition, maintains partial sums of propensities to enable a binary search for the reaction to be fired. The partial sum of propensities has to be re-computed from the point where the first partial sum has been changed for each reaction channel that is affected. The Sorting Direct Method (SDM) continually reorders the list of propensities to reduce search cost. Both of these methods are marginally better than the ODM, based on the model being simulated. The Composition Reaction (CR) method reduces the reaction search process to constant time [14]. The PSSA-CR method is the fastest variant of ODM to date. It uses propensity factorization to significantly reduce the cost of recomputing propensities of the affected reactions. Propensity factorization naturally distributes reactions into groups that share reactants while maintaining group propensity sum. Group propensity sums speed up the search for the next reaction to be fired. Additionally, the PSSA-CR method uses the composition rejection method to reduce search complexity. However, this method has complex update processes that involve significant amounts of pointer chasing, which is extremely difficult to parallelize (fine-level parallelization), especially on data-parallel architectures such as GPUs.

## Methods

In this paper, we describe a new variant of the Optimized Direct Method that is amenable to fine-level parallelization on GPUs. We present techniques to simultaneously achieve coarse-grained as well as fine-grained parallelism. We use threads within a warp to parallelize a single realization (fine-grained parallelization). Several warps executing simultaneously (coarse-grained parallelization) enable the simulation of several trajectories concurrently. Our implementation consists of three main phases. The first phase computes the index of the reaction to be fired and updates the state vector. The second phase updates the partial sums of the reaction blocks. The third phase checks to see if all realizations have completed their execution and terminates the program.

### Finding the index of the reaction to be fired

Finding the index of the reaction to be fired is the most expensive part as it involves a lot of memory transactions. Our method speeds up this step by significantly reducing memory transactions. The key idea is to divide the list of reaction propensities into blocks. The number of blocks is dependent on the total number of reactions. The size of the blocks is either a multiple of 128 or 256 reaction propensities (multiples of warp size). We keep track of the partial sum of propensities of each of these blocks through incremental updates. When the simulation begins, the block partial sums are computed from scratch on the CPU and uploaded to the GPU. Naturally this is a one time computation for all realizations. The total sum of propensities *a_R_* is the last entry in the list of block partial sums.

**Figure 2:**
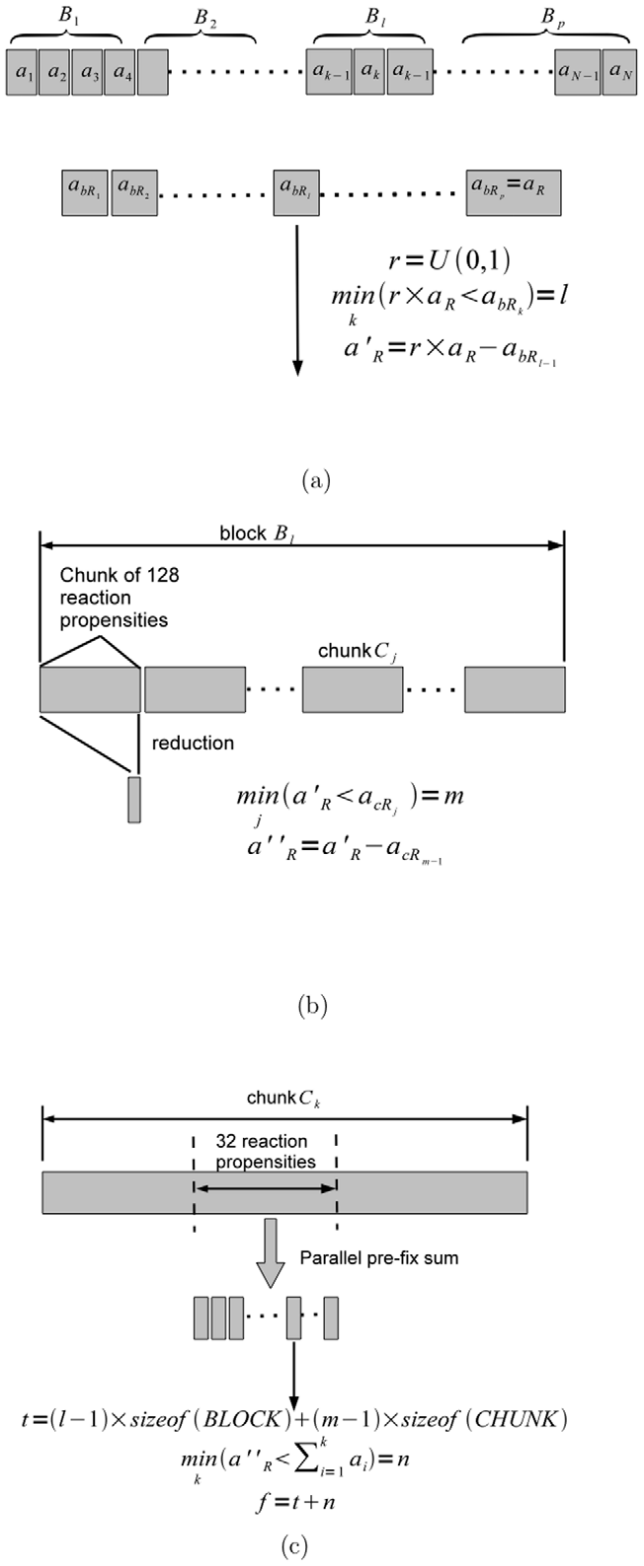
Three Level Search for Finding Index *f* of the Reaction to be Fired. In Figure 2(a), we use a warp voting function to find the block *B_l_* in which *r_f_* occurs. The cumulative block sums 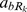 are maintained through incremental updates during simulation. In Figure 2(b), we use reduction and warp voting functions to find the chunk *C_m_* in block *B_l_* in which *r_f_* occurs. Finally in Figure 2(c), we use a parallel prefix sum and warp voting to find the index of *r_f_* in chunk *C_m_*.

We use a 3-level search with increasing granularity, and, consequently computation cost (Figure 2). We use a warp ballot (_ballot()) and first find set (_ffs()) functions in this search. The ballot function _ballot() sets the bits of a 32 bit integer based on the computed value of a predicate, one bit for each thread in the warp. The _ffs() function identifies the index or position of the least significant bit set to one in the 32 bit integer.

At the top level, we eliminate whole blocks through a parallel search. Given the product *z*_1_ × *a_R_*, each thread in the warp computes the predicate 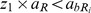, where 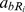 is the *i^th^* cumulative block sum of reaction propensities. The _ballot() function then fills a 32 bit integer based on this predicate. Next the _ffs() function is used to find 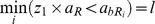, which is the index of the block where the reaction to be fired *r_f_* is located (Figure 2a).

Within a selected block *B_l_* we execute a search on chunks of 128 reactions at a time using warp-level reduction (Figure 2b). The reduction operation computes the cumulate chunk sum 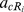 at the *i^th^* chunk. Each thread in a warp computes the sums of 128/32 = 4 propensities, each offset in memory with a stride of 32. Finally, a parallel-prefix sum is executed. This is essentially a coarse-level parallel linear search. Due to efficient use of shared memory and the opportunities for instruction-level parallelism, this step is very fast. When the chunk *C_j_* that contains the reaction *r_f_* to be fired next is found, we execute a fined-grained parallel search using a warp level pre-fix sum on the 128 reaction wide chunk in batches of 32 reaction propensities (Figure 2c). We first generate the partial sums of propensities for the 32 reaction propensities using a parallel pre-fix sum. Next, the _ballot() function is used to fill 32 bits of the integer based on the predicate 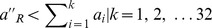. Finally, _ffs() returns the smallest index *k* where the predicate is true. The reaction corresponding to this index is *r_f_*.

### Updating

**Figure 3:**
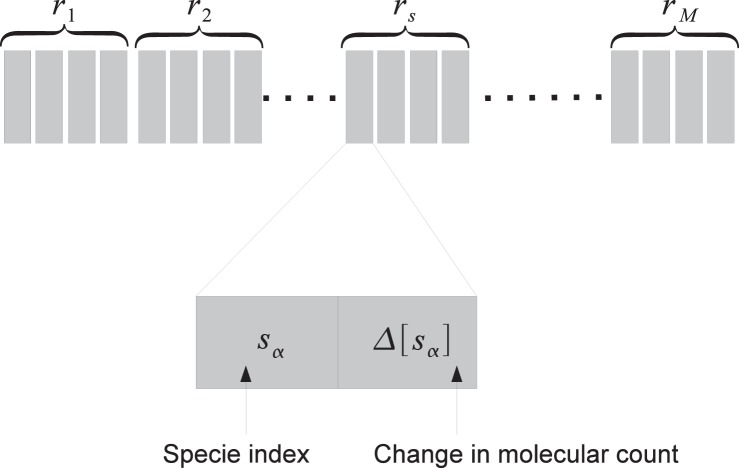
Stoichiometric Data Structure. The stoichiometric matrix is stored as a linear array. Since each reaction has at most 2 reactants and 2 products, each entry in the array (corresponding to one reaction) has 4 data fields. Each data field has 2 parts. The first is the index of the specie and the second is the change in the molecular count. The stoichiomteric data structure is common across all runs and therefore only a single copy is maintained in the global memory on the GPU.

The updating step involves moving the simulation time forward, updating populations of affected reactants, updating affected reaction propensities, and finally the updating block partial sums. We store the stoichiometric matrix in an array of int4 (since each reaction can have a maximum of two reactants and two products). Each int4 contains four integer elements. Each integer element contains the reactant identifier (first 29 bits) and the molecular change (last 3 bits) (Figure 3). This representation can handle 2^29^ reactants and all possible changes of molecular count (*v_ij_* ∈ [−2, −1, +1, +2]). Note that *v_ij_* is an element of the stoichiometric matrix and denotes the change in the molecular count of specie *s_i_* when reaction *r_j_* is fired. Therefore, given a reaction, it is very easy to update the molecular count. This is done by using at most four threads in the warp. When any reaction is fired, at most four species change molecular counts.

**Figure 4:**
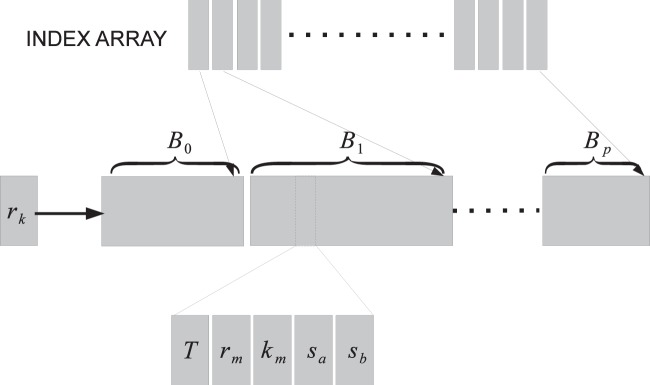
Reaction-Reaction Dependency Graph. Two arrays used to represent the dependency graph for each reaction *r_j_*. The first is an array of indices. The second is the array of dependent reactions sorted by the block in which they occur. The index array is used to indicate the end point for each block in the dependent reaction array. Each element of the dependent contains the type of the reaction *T*, the global index of the reaction *r_m_*, the reaction constant *k_m_*, and the indices of the reactant species *s_a_*, *s_b_*. The reaction-reaction dependency graph is common across all runs and therefore only a single copy is maintained in the global memory on the GPU.

Updating the block partial sums and the propensities is done simultaneously. We maintain in texture memory a reaction-reaction dependency graph (Figure 4). This data structure allows us to read the list of dependent reactions in one step in a coalesced manner at the cost of being inefficient in memory. Each node in this graph contains the reaction type (3 bits), reaction index (29 bits), the reaction rate (32 bits), and the indices of reactants (32 bits). The dependent reaction list of each reaction is sorted based on the location of the reactions in the blocks. An additional integer array stores the end index for each block in the sorted list of dependent reaction indices.

**Figure 5:**
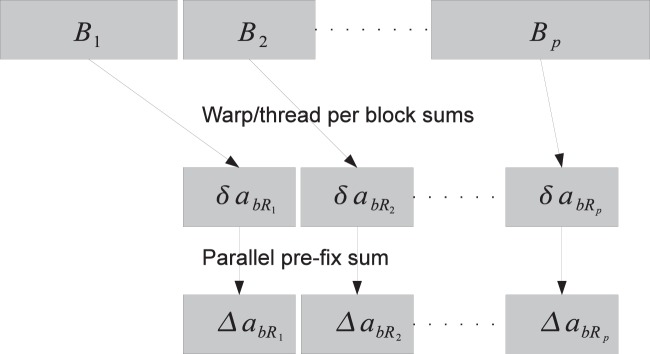
Updating Reaction Block Partial Sums. For sparsely connected systems, we use one thread per block to compute the change in the sum of propensities 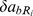, within a block *B_i_*. For densely connected systems, the whole thread warp is used. The changes in the partial block sums 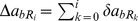 are found for all *i* = 1, 2…*p* in parallel using the parallel-prefix sum. Finally, the block partial propensity sums are updated in parallel as 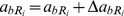.



For weakly connected reactions where there are on an average less than 32 (size of the warp) dependent reactions per block, we use one thread per block for processing. For every reaction belonging to a given block, the thread assigned to the block reads the old propensity from global memory, computes the new propensity using the updated molecular count of reactants (this was done in the previous step), writes the new propensity to global memory, and finally computes the change in propensity and stores it in shared memory in an temporary array. The size of this array is equal to the number of blocks. For strongly connected systems, where the average number of dependent reactions is greater than 32, we use the entire warp to process the dependent reactions in the block. We use a reduction operation where each thread processes multiple reactions and sums the change in propensities into a separate array shared memory (Figure 5). Finally, a parallel prefix sum generates the total change in block sums for every block. The entries in this array at this stage are 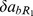, 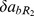, 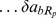. Since a reaction *r_k_* ∈ *B_l_* affects the blocks' sums of all blocks *B_m_*: *m* ≥ *l*, the block sum updates are 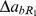, 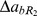, 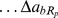, where 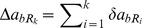. We obtain 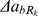 by executing a parallel-pre-fix sum on the array containing 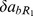, 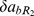, 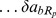. Next we can update all block sums in parallel as:

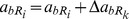



There is a trade-off between the number of blocks and the effort required to update block sums. Smaller block sizes imply a faster search of the index of the reaction to be fired but slower updates due to the large number of blocks and vice versa. The trade-off is dependent on the size of the problem as well as the interconnectivity.

### Pre-processing input data

In many biological reaction networks, a few reaction channels dominate the dynamics and are fired more often than others. This enables the speed of the simulation through clever placement of data in memory such that the cache utilization is maximized. We run a pre-simulation using the ODM and record the number of times a given reaction propensity and the specie's molecular count is accessed. We then sort both the array of the reaction propensities and the array of the species molecular counts, based on the number of accesses. This places the propensities of most the frequently fired reactions and their dependent reactions as well as the related species molecular counts in relative proximity in memory. Therefore, when a reaction is fired and the data structure is updated (i.e., molecular counts, block sums, and propensities of dependent reactions), the required data has a very high probability of being in cache, thus significantly reducing the memory transaction overhead.

## Results

We have benchmarked the performance of GPU-ODM on several models, namely, the cyclic chain model (weak dependencies) [5], the colloidal aggregation model (strong dependencies) [26], and using a randomly generated network (see File S1). We compared the performance of GPU-ODM with ODM, CR, SPDM, PSSA-CR. We compiled our code using gcc4.4 with appropriate optimization flags and executed it on an Intel i7-930 CPU (32 KB L1 cache, 1MB L2 cache (256KB per core), 8MB L3 cache, 52 GFlops) with 6GB of RAM. The operating system was Windows 7. The GPU part of the code was executed on a consumer grade NVIDIA 480GTX GPU.

**Figure 6:**
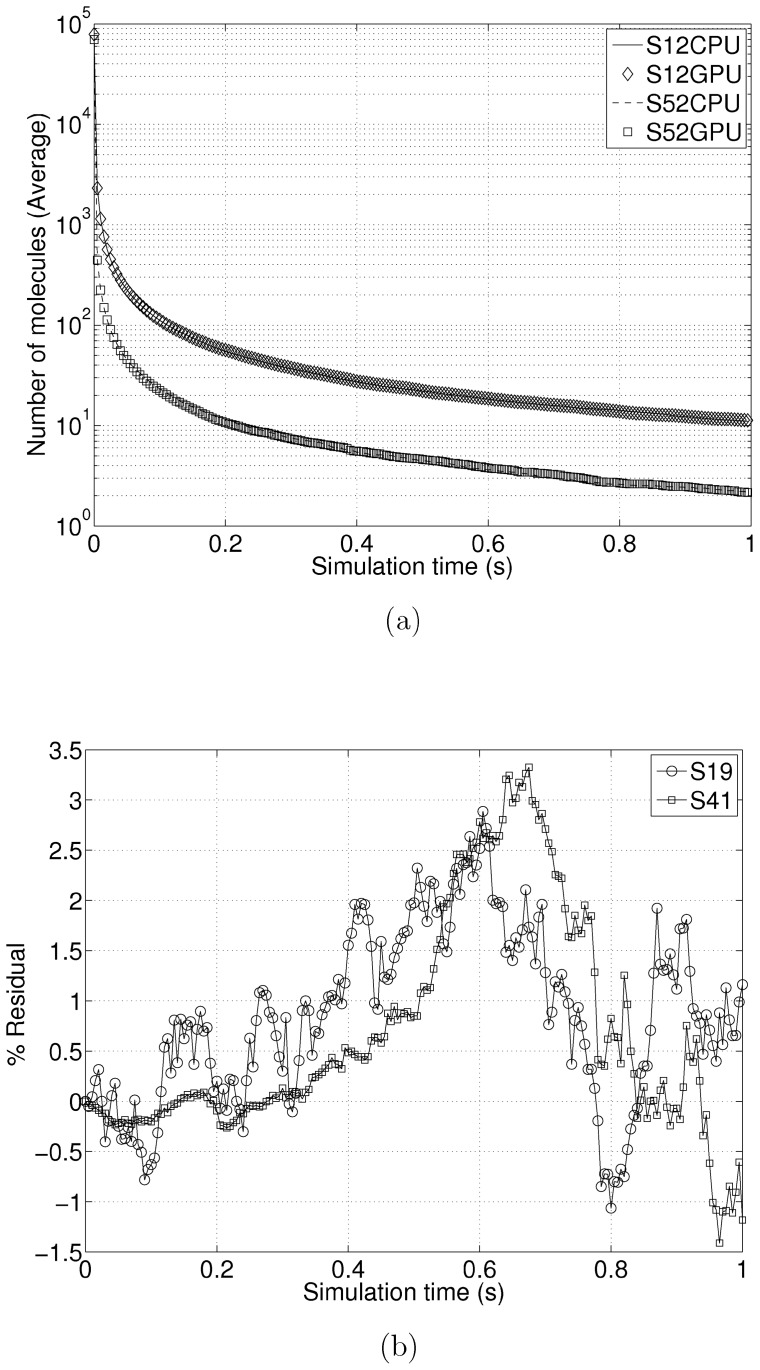
Accuracy Benchmarks. Figure 6(a) shows the time trajectories of two randomly selected species output from the GPU and CPU executions. Figure 6(b) shows the percentage residuals of time trajectories of two other randomly selected species.

We verified the accuracy of our implementation on a randomly generated network with 1024 reactions and 1024 species and tested the accuracy of our implementation against ODM in StochKit. We executed 10,000 independent trajectories and averaged the results. Since the time increments at each update step are different for different trajectories, we used linear interpolation to compute the molecular counts at predefined time intervals (0.01s). Figure 6(a) show the average trajectories for a few randomly selected species. Figure 6(b) shows the residual percentages at each time step for another set of randomly selected species. The percentage residual is <4%.

**Figure 7:**
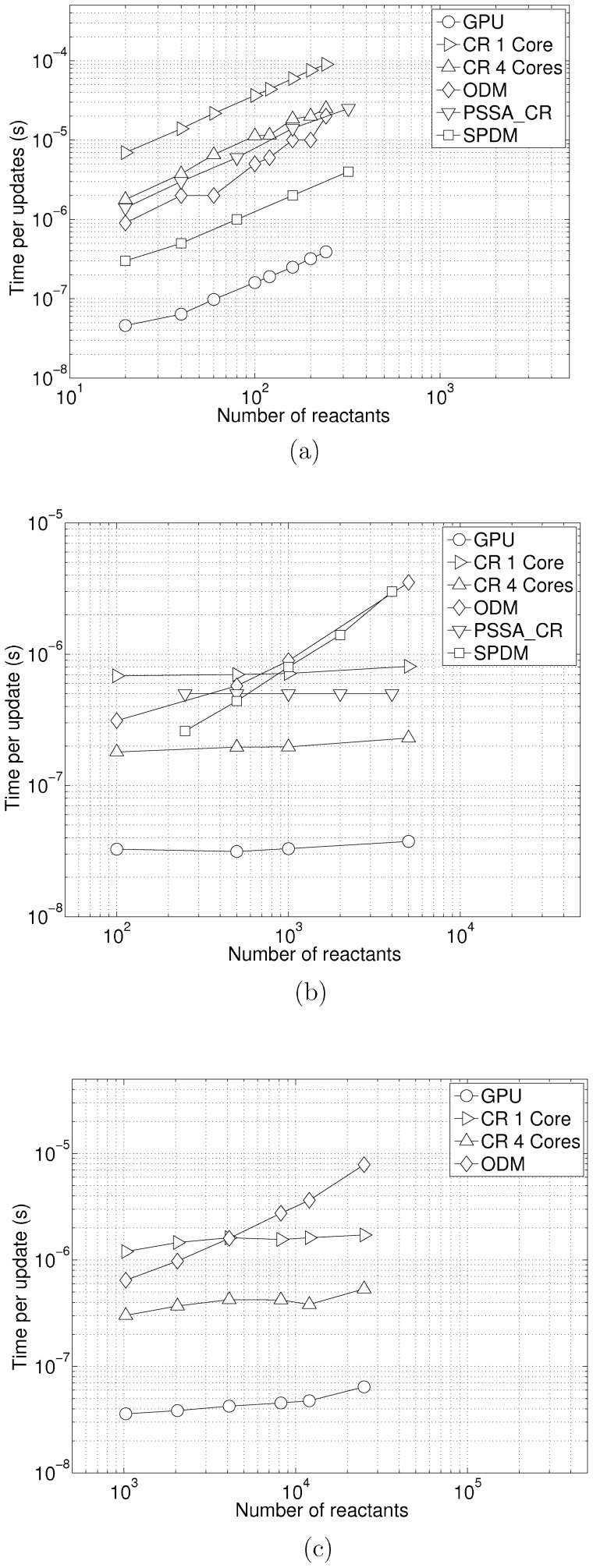
Performance Benchmarks. Figure 7(a) list shows the results for the Colloidal Aggregation Model (the strongly connected system). For *N* chemical species, the number reactions is 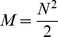. The number of dependent reactions is 3*N*–7 and therefore scales with the number of species. Figure 7(b) shows the results for the Cyclic Chain Model (the weakly connected system). For *N* chemical species, this network has *M* = *N* reactions. The initial molecular counts of all species [*s_i_*] were set to 1. All reaction constants *k_i_* were set to 1. These initial conditions are the same as in [13]. The graphs for PSSA-CR and SPDM were obtained from [13]. Figure 7(c) shows the results for the randomly generated system. The size of the dependent reactions list varies for 8–16 for these systems. Note that running the CR algorithm on all 4 cores (one realization per core) gives a performance gain <4× over a single core run. This gain could be expected of all other serial CPU algorithms as well.

We conducted three performance tests. The time per update is computed as:
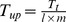
where *T_up_* is the time per update, *T_t_* is the total computation time on the GPU neglecting pre-simulation, *l* is the number of realizations, and *m* is the number of average updates. We ran 5000–10000 realizations with about 10000–50000 updates for various models. Figure 7a illustrates the time per update for the cyclic chain model with respect to the number of reactants (which, in this case, is equal to the number of reactions as well). GPU-ODM outperforms the best serial implementation (PSSA-CR) [13] by about 10× for systems that fit our GPU memory. Figure 7b illustrates the time per update for the colloidal aggregation model. Our GPU-ODM outperforms SPDM [13] by about 8× for large systems. The PSSA-CR and SPDM algorithms were written in C++ and run on a Linux 2.6 workstation with a 2.8 GHz quad-core Intel Xeon E5462 processor (32 KB L1 cache, 12 MB of L2 cache, 44.8 GFlops) and 8GB of RAM memory. This processor is comparable to our i7-930, perhaps slightly better for random memory access with the larger L2 cache. Figure 7c illustrates the time per update for a random model. The dependencies here are between the cyclic chain model and the colloidal aggregation model. GPU-ODM outperforms the CR method by about 26×.

**Figure 8:**
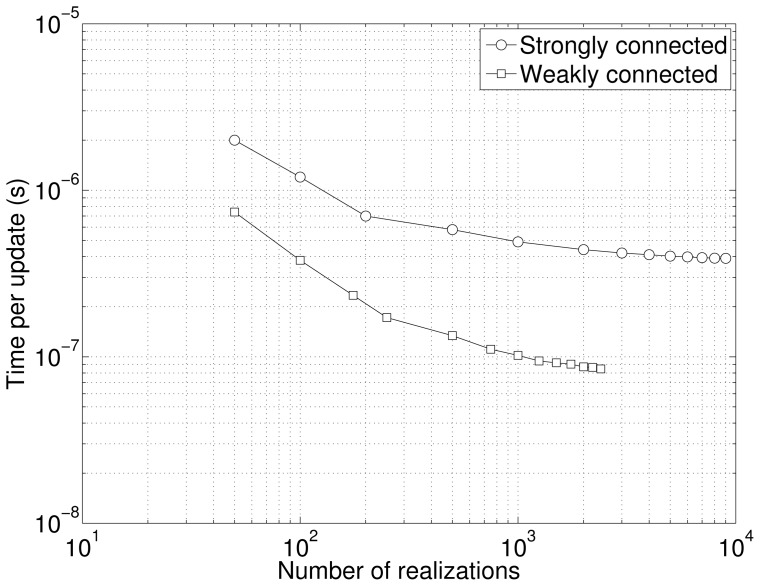
Performance w.r.t. Number of Realizations. Both the strongly connected and the weakly connected systems show a decrease in time per update as the number of realizations is increased. This decrease flattens out, indicating saturation of the GPU computing power. The strongly connected system here had the number of species at *N* = 252 and the weakly connected system had *N* = 50, 000.

We also tested the performance of our implementation with respect to the number of realizations. As noted before, the GPU delivers lower performance if there is an insufficient number of realizations due to underutilization of the computing resources. Figure 8 illustrates the time per update vs. the number of realizations for both the colloidal aggregation model as well as the cyclic chain model. Both systems exhibit the same behavior with a drastic drop in time per update until about 1000 realizations.

**Figure 9:**
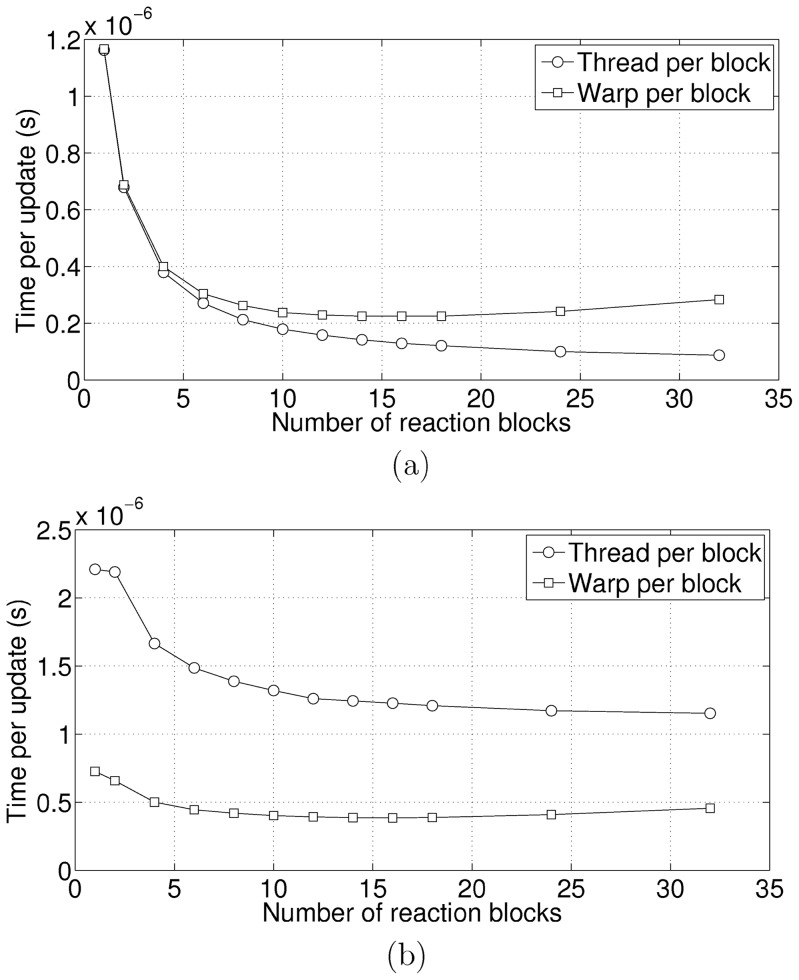
Performance w.r.t. Number of Reaction Blocks. Figure 9(a) shows the results for a weakly connected system. Figure 9(b) shows the results for a strongly connected system. Note that in both instances, the warp per block performance initially increases with the number of blocks and then decreases. This is because the warp per block includes a parallel-prefix sum for each reaction block that can get expensive.

Finally, we tested the performance with respect to the number of reaction blocks. As explained earlier, a larger number of blocks reduces the complexity of the search process for finding the reaction to be fired but increases the update complexity. Therefore, there is a sweet spot where the time is minimum. The models that we tested were the cyclic chain model (weakly connected) and the colloidal aggregation model (strongly connected). We tested two variants of our update algorithm, one with warp per block and one with thread per block. For the warp per block, the sweet spot is about 16 reaction blocks (Figure 9(a)). In the case of the thread block, there is a significant reduction in time per update initially with the graph flatening out at about 25 reaction blocks (Figure 9(b)). It is evident that for a small number of block sizes, the processing time is dominated by the search for the reaction to be fired. In the case of the cyclic chain model, since there are only 2 dependencies per reaction, the cost of updating block partial sums is essentially constant (one 32 element wide parallel prefix sum). In the case of the warp per block, as the number of reaction blocks increases, the cost also increases because there is a parallel prefix sum per block used in the update.

## Discussion

We have successfully implemented a hybrid (coarse and fine grained) parallel implementation of a new exact Gillespie SSA variant on the GPU. We have verified the correctness of our implementation by benchmarking against StochKit.

Unlike previous parallel GPU implementations, the size of the network being simulated is not limited by on-chip shared memory available to a single thread, but is limited by DRAM, which is orders of magnitude larger than shared memory. More importantly, it is easier to increase the amount of DRAM as opposed to on-chip shared memory. In the near future, when GPU and CPUs will be fused with GPUs having direct access to the system DRAM, the network sizes that our implementation will be able to handle will grow much larger. Given a fixed amount of DRAM, there is a trade-off between the size of the network being simulated and the number of trajectories that can be generated simultaneously. This also has an impact of utilization. A large network can consume so much memory so as to limit the number of realizations to a level where all hardware resources on the GPU cannot be used.

Gillespie SSA implementations in general are data-bound, i.e., a majority of the time is spent in moving data between DRAM and the processor. The order of placement of reaction propensities in global memory affects the performance of the algorithm. A typical memory transaction takes about 300 cycles (as opposed to 20 cycles for division) on the GPU. Furthermore, if the location of the global memory being addressed by each thread is randomly distributed, as is typically the case with stochastic algorithms such as the Gillespie SSA, it can result in multiple memory transactions, thus affecting performance. In our implementation, we have traded data redundancy for performance. For example, the reaction dependency graph carries a portion of the stoichiometric data (reactant indices). Furthermore, if a reaction appears as a dependent reaction in more than one list, these data are repeated. A second optimization is by clever placement of data in DRAM to maximize cache utilization. Our scheme of sorting the reaction propensities and reactant molecular count lists based on pre-simulation has the effect of improving performance by about 30%–40%. Since the cost of pre-simulation is done once for all the realizations, the cost of pre-simulation amortized over thousands of runs is negligible and is not counted.

A major limitation in our implementation is the reaction-reaction dependency graph. Large, tightly coupled systems can overwhelm memory if a simple dependency graph is used. Due to the nature of tightly coupled bio-chemical systems, where a relatively low number of species is involved in a large number of reactions, a bi-partite graph can significantly reduce the amount of memory needed to store reaction dependencies. However, a bi-partite graph necessitates an extra irregular memory indirection that could greatly affect performance. For typical systems that we simulated using our implementations, the reaction-reaction dependency graph's memory consumption is not a major issue. We could possibly use a bi-partitie graph to reduce the memory footprint at the cost of performance.

Finally, we conclude that the performance of any algorithm is highly dependent on the nature of the system being simulated. Factors such as system interconnectivity, network size, and relative dominance of reaction channels in the dynamics all significantly affect performance. This is the main reason why we see a drastic performance difference while simulating different systems with the same algorithm. Overall, our GPU-ODM performs much better than the state-of-the-art algorithms across the board. Given that our implementation runs on hardware that has about a 100X advantage in theoretical computing power, one can clearly see that in certain instances, our algorithm greatly underutilizes the available resources.

## Supporting Information

File S1 **Generating Random Consistent Synthetic Networks.**
(PDF)
